# Diagnostic Value, Prognostic Value, and Immune Infiltration of *LOX* Family Members in Liver Cancer: Bioinformatic Analysis

**DOI:** 10.3389/fonc.2022.843880

**Published:** 2022-03-04

**Authors:** Chenyu Sun, Shaodi Ma, Yue Chen, Na Hyun Kim, Sujatha Kailas, Yichen Wang, Wenchao Gu, Yisheng Chen, John Pocholo W. Tuason, Chandur Bhan, Nikitha Manem, Yuting Huang, Ce Cheng, Zhen Zhou, Qin Zhou, Yanzhe Zhu

**Affiliations:** ^1^ AMITA Health Saint Joseph Hospital Chicago, Chicago, IL, United States; ^2^ Department of Epidemiology and Health Statistics, School of Public Health, Anhui Medical University, Hefei, China; ^3^ Department of Clinical Medicine, School of the First Clinical Medicine, Anhui Medical University, Hefei, China; ^4^ Gastroenterology, AMITA Health Saint Joseph Hospital Chicago, Chicago, IL, United States; ^5^ Mercy Internal Medicine Service, Trinity Health of New England, Springfield, MA, United States; ^6^ Department of Diagnostic Radiology and Nuclear Medicine, Gunma University Graduate School of Medicine, Maebashi, Japan; ^7^ Department of Orthopedics, Shanghai General Hospital, Shanghai Jiao Tong University School of Medicine, Shanghai Jiao Tong University, Shanghai, China; ^8^ University of Maryland Medical Center Midtown Campus, Baltimore, MD, United States; ^9^ College of Medicine, The University of Arizona, Tucson, AZ, United States; ^10^ Banner-University Medical Center South, Tucson, AZ, United States; ^11^ Menzies Institute for Medical Research, University of Tasmania, Hobart, TAS, Australia; ^12^ Department of Radiation Oncology, Mayo Clinic, Rochester, MN, United States; ^13^ Department of Oncology, The First Affiliated Hospital of Anhui Medical University, Hefei, China

**Keywords:** liver cancer, lysyl oxidase, bioinformatic analysis, receiver operating curve, nomogram, prognostic value, immune infiltration

## Abstract

**Background:**

Liver cancer (LC) is well known for its prevalence as well as its poor prognosis. The aberrant expression of lysyl oxidase (*LOX*) family is associated with liver cancer, but their function and prognostic value in LC remain largely unclear. This study aimed to explore the function and prognostic value of LOX family in LC through bioinformatics analysis and meta-analysis.

**Results:**

The expression levels of all *LOX* family members were significantly increased in LC. Area under the receiver operating characteristic curve (AUC) of *LOXL2* was 0.946 with positive predictive value (PPV) of 0.994. *LOX* and *LOXL3* were correlated with worse prognosis. Meta-analysis also validated effect of *LOX* on prognosis. Nomogram of these two genes and other predictors was also plotted. There was insufficient data from original studies to conduct meta-analysis on *LOXL3*. The functions of *LOX* family members in LC were mostly involved in extracellular and functions and structures. The expressions of *LOX* family members strongly correlated with various immune infiltrating cells and immunomodulators in LC.

**Conclusions:**

For LC patients, *LOXL2* may be a potential diagnostic biomarker, while *LOX* and *LOXL3* have potential prognostic and therapeutic values. Positive correlation between *LOX* family and infiltration of various immune cells and immunomodulators suggests the need for exploration of their roles in the tumor microenvironment and for potential immunotherapeutic to target *LOX* family proteins.

## Background

Liver cancer (LC) is the sixth most common malignant tumor and the third leading cause of cancer-associated mortality worldwide. Hepatocellular carcinoma (HCC) accounts for 75%-85% of all LC, according to the GLOBOCAN 2020 estimation (1). In east Asia, especially China, a high incidence of HCC was noted, and similarly, the incidence and mortality of LC in developing countries are significantly higher than those in developed countries (2, 3). The variations in the prevalence of LC amongst different populations and regions are attributed to a variety of environmental and genetic factors, such as aflatoxin, alcohol, smoking, chronic hepatitis virus infection, and type 2 diabetes (4–6). Despite significant advances in diagnosis and treatment of LC, including surgical resection, local ablation, liver transplantation, and sorafenib–regorafenib sequential therapy, the prognosis of LC remains poor (6). Based on most recent data, 841,000 new cases and 782,000 deaths of LC around the globe was estimated to occur each year (1). Therefore, it is of great value to explore novel diagnostic and prognostic biomarkers that are sensitive and specific, and to identify potential targets for medications (7).

With the advent of next-generation sequencing (NGS) and other techniques, increasing amount of information has become available for a variety of cancer types and other diseases (8–11). Thus, the mechanisms of cancers, as well as other diseases, have become more widely investigated based on bioinformatic methods, by combining information technology and molecular biology. Bioinformatics methods, such as data-mining, are also widely applied for identification of potential biomarkers as therapeutic targets, as well as diagnostic and prognostic predictors, and to explore the pathogenesis of malignancies at the molecular level (12–14).

As an extracellular enzyme, lysyl oxidase (*LOX*) oxidatively deaminates specific lysine and hydroxylysine residues to form allysines in the telopeptide domains of the collagen molecule, and thus plays a critical role in covalent cross-link formation in collagen fibrils (15). *LOX* is highly expressed in tissues containing elastic fibers and fibrillar collagen, such as skin, lung, and the fibrous lamina propria in the small intestine, stomach, and liver (16). In addition to *LOX*, four *LOX*-like proteins (*LOXL*-1, -2, -3, and -4) have also been identified in the *LOX* family (17–19). Studies have found that the *LOX* family was involved in carcinogenesis and tumor metastasis, through angiogenesis promotion, formation of mature extracellular matrix at the secondary site, focal adhesion kinase (FAK) activation, and other mechanisms (19–22).

The lysyl oxidase (*LOX*) family consists of five members: *LOX*, the first described member of this family, and its four related members called lysyl oxidase-like genes (*LOXL1-4*). Recent evidence suggests that the *LOX* family play important roles in liver cancer. *LOX* secreted by HCC promotes tube formation of endothelial cells through upregulation of VEGF, and overexpressed *LOX* increases angiogenesis, whereas *LOXL1* was found to be increased in liver fibrosis models (23–25). As for *LOXL2*, its expression level was found to be higher in HCC tissues compared with non-tumor tissue (26). Although *LOXL3* has been studied in different types of cancer, studies on its roles in liver cancer are limited (25). *LOXL4* was found to increase the risk of invasion and metastasis, promote angiogenesis, and play a role in the immunosuppressive microenvironment in HCC (25, 27, 28)

Although previous studies have investigated the roles of the *LOX f*amily in LC, their exact roles and mechanisms, especially for *LOXL1* and *LOXL3*, have yet to be further investigated (25). Previous studies have shown evidence of the potential prognostic values and therapeutic values of *LOX* family members (25, 26). Thus, online databases were mined to analyze the expression, mutation, function, and immune infiltration of *LOX f*amily members in LC, with the goal to determine their potential oncogenic role, as well as their diagnostic and prognostic value in LC.

## Results

### Differential Expression Levels of *LOX* Family in LC

**Figure 1:**
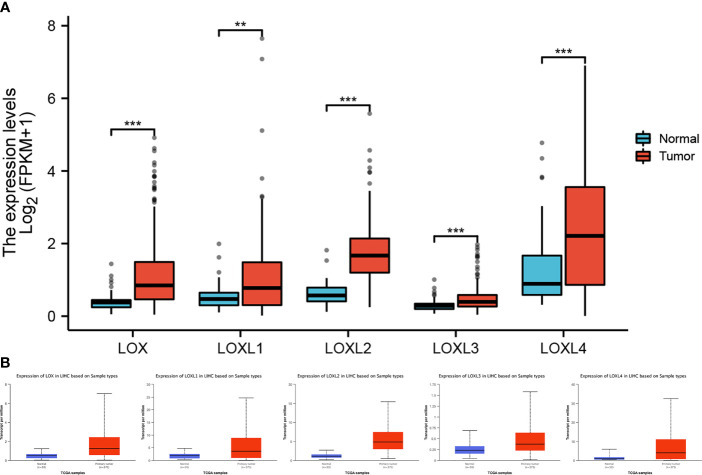
Expression level of *LOX* family members between normal tissue and tumor tissue in liver cancer. **(A)** analysis *via* R software, **(B)** analysis *via* ULCAN. ** means P < 0.01, *** means P < 0.001.

**Figure 2:**
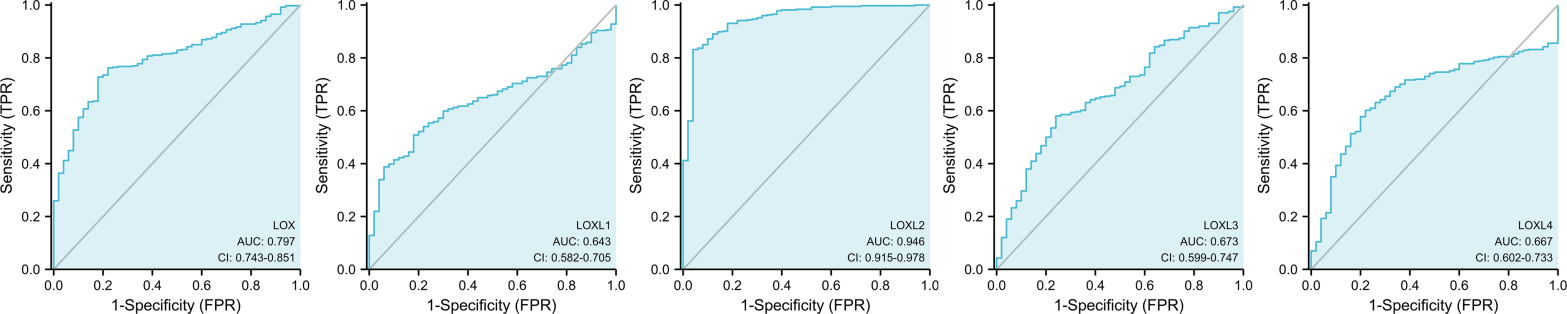
ROC curve analysis for *LOX* family members in liver cancer.

All five members of *LOX* family demonstrated higher expression in liver cancer tumor tissues than normal tissues (**Figure 1A** and **Table 1**). These findings were consistent with results from UALCAN, which confirmed that the expression of all *LOX* family members was statistically significantly higher in tumor tissue (**Figure 1B**), and from TIMER, which showed higher expression of *LOX* (P=1.5E-11), *LOXL1* (P=2.39E-04), *LOXL2* (P=4.02E-25)*, LOXL3* (P=1.53E-04), and *LOXL4* (P=7.15-05). Further analysis of ROC curve showed that AUC of *LOXL2* was 0.946 (95%CI:0.915-0.978, with positive predictive value (PPV) of 0.994 and a cutoff value of 1.050 (**Figure 2**).

**Table 1:** Expression level of *LOX* family members between normal tissue and tumor tissue in liver cancer, and overall survival of overexpressing *LOX* family members in liver cancer.

Gene name	Mean			TCGA			TIMER		
	Normal tissue group	Tumor tissue group	P value	HR	95%CI	P value	HR	95%CI	P value
*LOX*	0.418 ± 0.268	1.112 ± 0.898	< 0.001	1.53	1.08-2.16	0.017	1.223	1.073-1.416	0.003
*LOXL1*	0.540 ± 0.381	1.018 ± 0.944	= 0.001	0.93	0.66-1.32	0.693	0.999	0.897-1.113	0.986
*LOXL2*	0.623 ± 0.311	1.725 ± 0.735	< 0.001	1.19	0.84-1.68	0.333	1.188	0.984-1.434	0.073
*LOXL3*	0.312 ± 0.178	0.463 ± 0.300	< 0.001	1.65	1.16-2.35	0.005	1.524	1.059-2.194	0.023
*LOXL4 *	1.307 ± 1.092	2.300 ± 1.625	< 0.001	1.44	1.02-2.05	0.038	1.080	0.980-1.191	0.119

### Prognostic Value of *LOX* Family in LC

**Figure 3:**
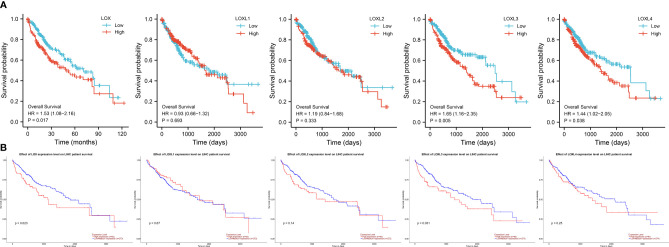
Survival analysis of *LOX* family members in liver cancer. **(A)** analysis *via* R software, **(B)** analysis *via* ULCAN.

Evaluation of the value of differential expression of *LOX* family members in LC prognosis found that *LOX*, *LOXL3*, and *LOXL4* were associated with poor overall survival (OS) (**Figure 3A** and **Table 1**). UALCAN was utilized for verification which found that only *LOX* (P=0.023) and *LOXL3* (P=0.031) were associated with poor OS (**Figure 3B**). Further verification *via* TIMER also only identified poor prognosis of *LOX* (P=0.003) and *LOXL3* (P=0.023) (**Table 1**). The combined results indicated that high expression of *LOX* and *LOXL3* was associated with worse OS.

**Figure 4:**
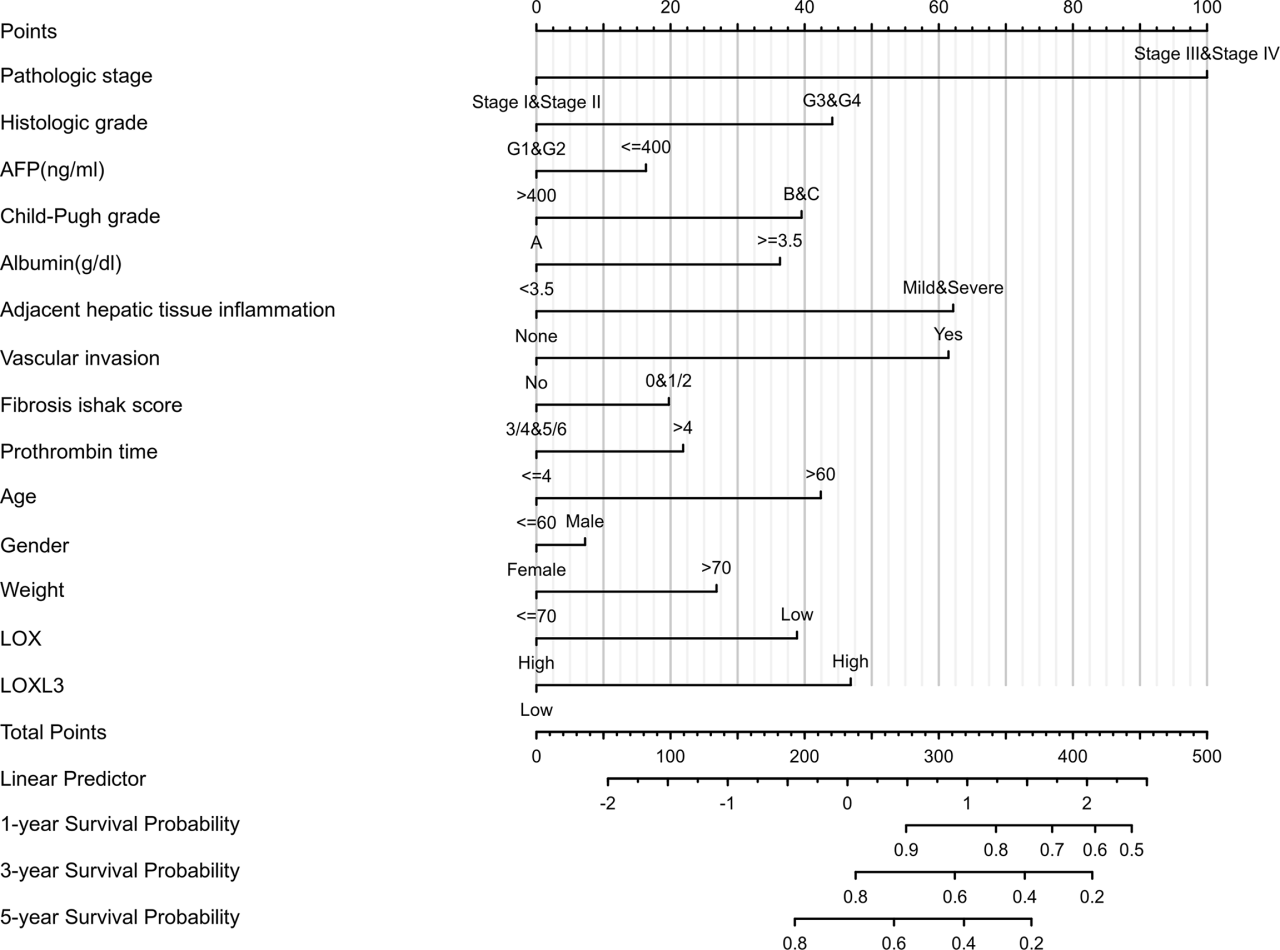
Nomogram for liver cancer based on overexpressed *LOX* and *LOXL3*. The nomogram was developed in the cohort, with pathologic stage, histologic grade, AFP (ng/ml), Child-Pugh grade, albumin (g/dl), adjacent hepatic tissue inflammation, vascular invasion, Ishak Fibrosis score, prothrombin time, age, gender, weight. (C-index: 0.738, 95% CI, 0.697-0.778).

A nomogram model incorporating the overexpressed *LOX* family members that were associated with poor prognosis, namely *LOX* and *LOXL3* and other predictors (pathologic stage, histologic grade, AFP (ng/ml), Child-Pugh grade, albumin (g/dl), adjacent hepatic tissue inflammation, vascular invasion, Ishak Fibrosis score, prothrombin time, age, gender, weight) is shown in **Figure 4**. The C-index of the nomogram was 0.738 (95% CI, 0.697-0.778).

### Analysis of Genetic Mutations of *LOX* Family in LC

**Figure 5:**
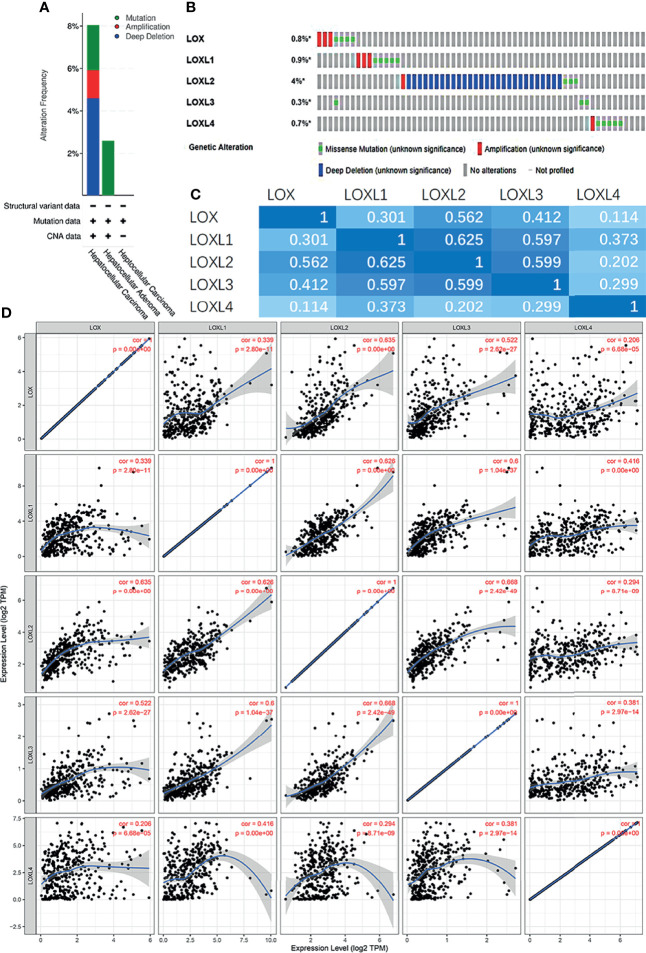
Gene mutation and expression analysis of *LOX* family members in patients with liver cancer: **(A)** Genetic alterations of *LOX* family members in different histopathologic types of liver cancer; **(B)** Summary of genetic alterations in different expressed *LOX* family members in liver cancer; **(C)** Correction between different *LOX* family members in in liver cancer (cBioPortal); **(D)** Correction between different *LOX* family members in in liver cancer (TIMER).

Next, the genetic alterations of the *LOX* family in LC patients were evaluated with the cBioPortal online tool. Among 1,066 LC patients, 55 samples had genetic alteration of *LOX* family members, with a mutation rate of 5.16%. The mutation rate of *LOXL2* was the highest (4%) (**Figures 5A, B**). Using cBioPortal and TIMER online tools, we found significant (p<0.01) and positive correlations amongst *LOX* family member proteins: *LOX* with *LOXL1*, *LOXL2*, *LOXL3*, and *LOXL4*; *LOXL1* with *LOX*, *LOXL2*, *LOXL3*, and *LOXL4*; *LOXL2* with *LOX*, *LOXL1*, *LOXL3*, and *LOXL4*; *LOX3* with *LOX*, *LOXL1*, *LOXL2*, and *LOXL4*; *LOX4* with *LOX*, *LOXL1*, *LOXL2*, and *LOXL3* (**Figures 5C, D**).

### Exploration of Potential Drugs That Are Interacted With *LOX* Family Members in LC

**Figure 6:**
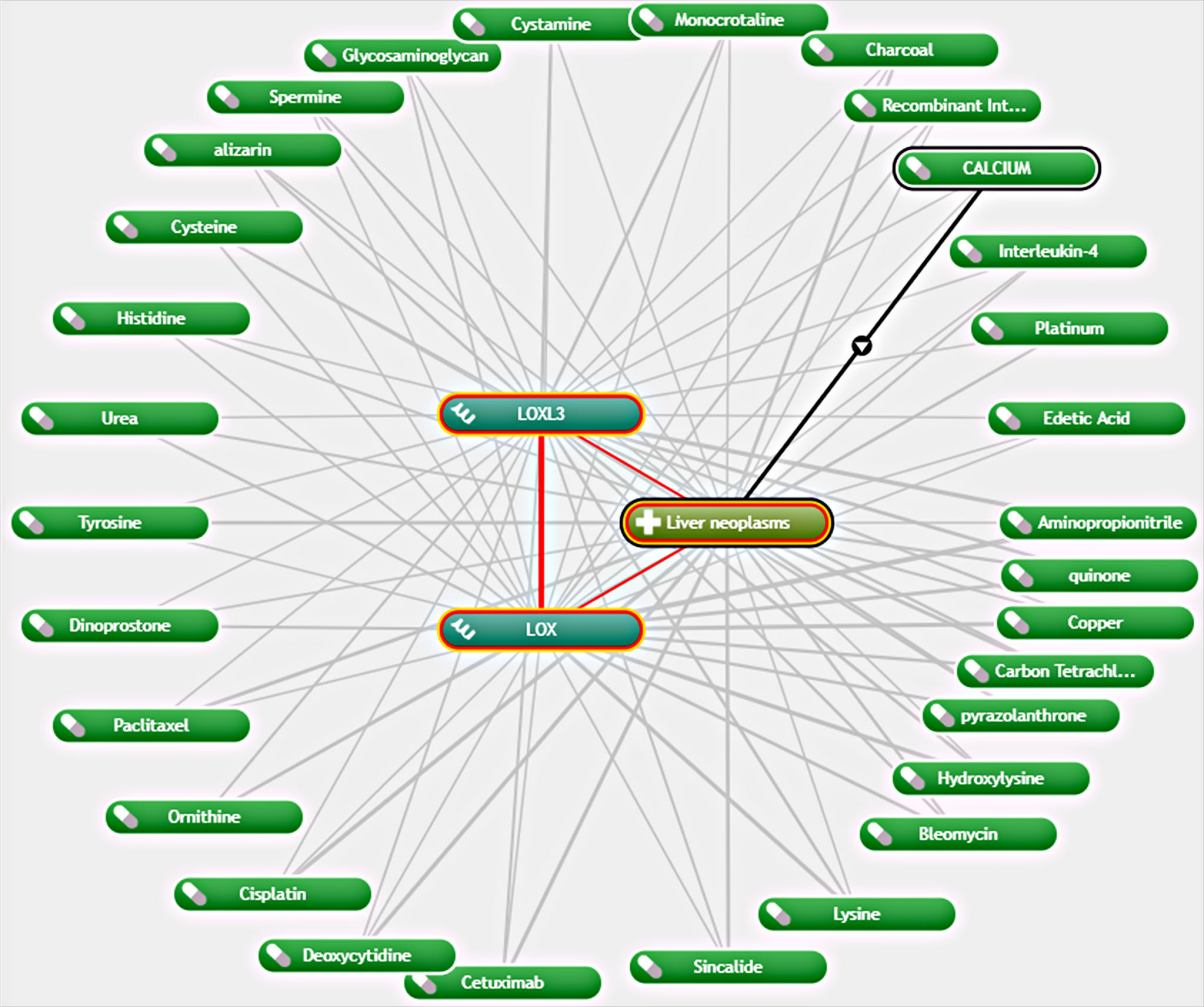
Network of association between *LOX* and *LOXL3* and different drugs in liver neoplasm *via* Coremine Medical.

As *LOX* and *LOXL3* were both found to be overexpressed in LC and associated with worse OS, further exploration of potential interacting drugs was conducted by using Coremine Medical, which identified 30 drugs that were associated with both *LOX* and *LOXL3* in liver neoplasms (**Figure 6**). The top three drugs were Aminopropionitrile, quinone, and copper.

### Analysis of Interaction of *LOX* Family Members in Patients With LC

**Figure 7:**
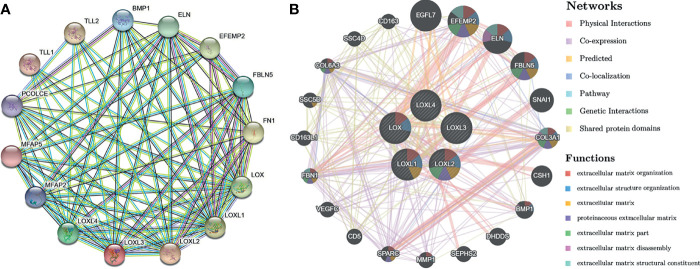
Protein-protein interaction (PPI) network analysis of *LOX* family members in patients with liver cancer. **(A)** PPI network of *LOX* family members and their interactors visualized by STRING; **(B)** PPI network of *LOX* family members and their interactors visualized by GeneMANIA.

Using the STRING database, PPI network analysis was performed on the differentially expressed *LOX* family members and 10 proteins (*BMP1, ELN, EFEMP2, FBLN5, FN1, MFAP2, MFAP5, PCOLCE, TLL1, TLL2*) that significantly interacted with *LOX* family members to further explore their potential interactions (**Figure 7A**). The results from GeneMANIA also revealed the function of differentially expressed *LOX* family members. Their top 20 associated interactors were primarily related to extracellular matrix organization, extracellular structure organization, extracellular matrix, proteinaceous extracellular matrix, extracellular matrix part, extracellular matrix disassembly, and extracellular matrix structural constituent (**Figure 7B**).

### GO Enrichment and KEGG Pathway Analysis of *LOX* Family Members in LC

**Figure 8:**
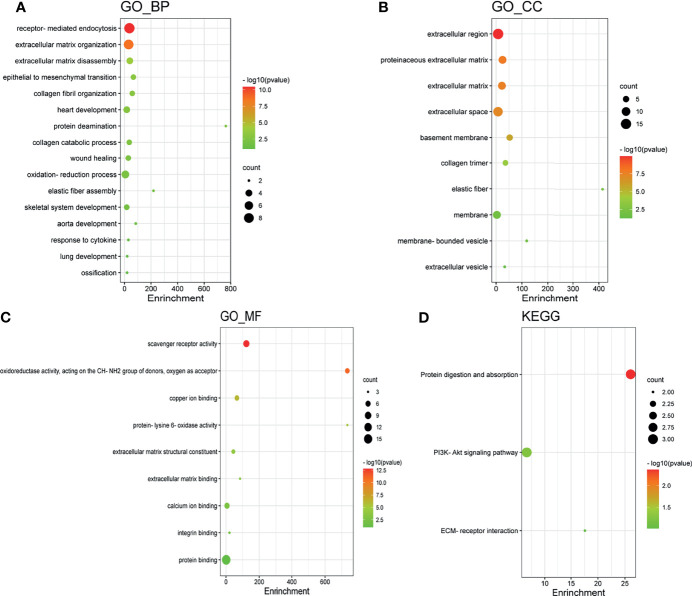
Gene Ontology (GO) enrichment and Kyoto Encyclopedia of Genes and Genomes (KEGG) pathway analysis of *LOX* family members and their interactors. GO enrichment analysis of target genes based on **(A)** biological process, **(B)** cellular component, and **(C)** molecular function. **(D)** KEGG pathway enrichment analysis of target genes.

GO enrichment and KEGG pathway analysis of *LOX* family members and their 20 interactors were conducted by using DAVID. Receptor-mediated endocytosis, extracellular matrix organization, and extracellular matrix disassembly were the top three biological processes that were associated with *LOX* family members and their interactors (**Figure 8A**). The extracellular region, proteinaceous extracellular matrix, and extracellular matrix were the top three major cellular components of the target genes (**Figure 8B**). As for molecular function, scavenger receptor activity, oxidoreductase activity (acting on the CH-NH2 group of donors, oxygen as acceptor), and copper ion binding were the top three functions (**Figure 8C**). In regard to KEGG pathways, protein digestion and absorption, PI3K-Akt signaling pathway, and ECM-receptor interaction were the top three pathways involved in LC (**Figure 8D**).

### Immune Cell Infiltration of *LOX* Family Members in LC

**Figure 9:**
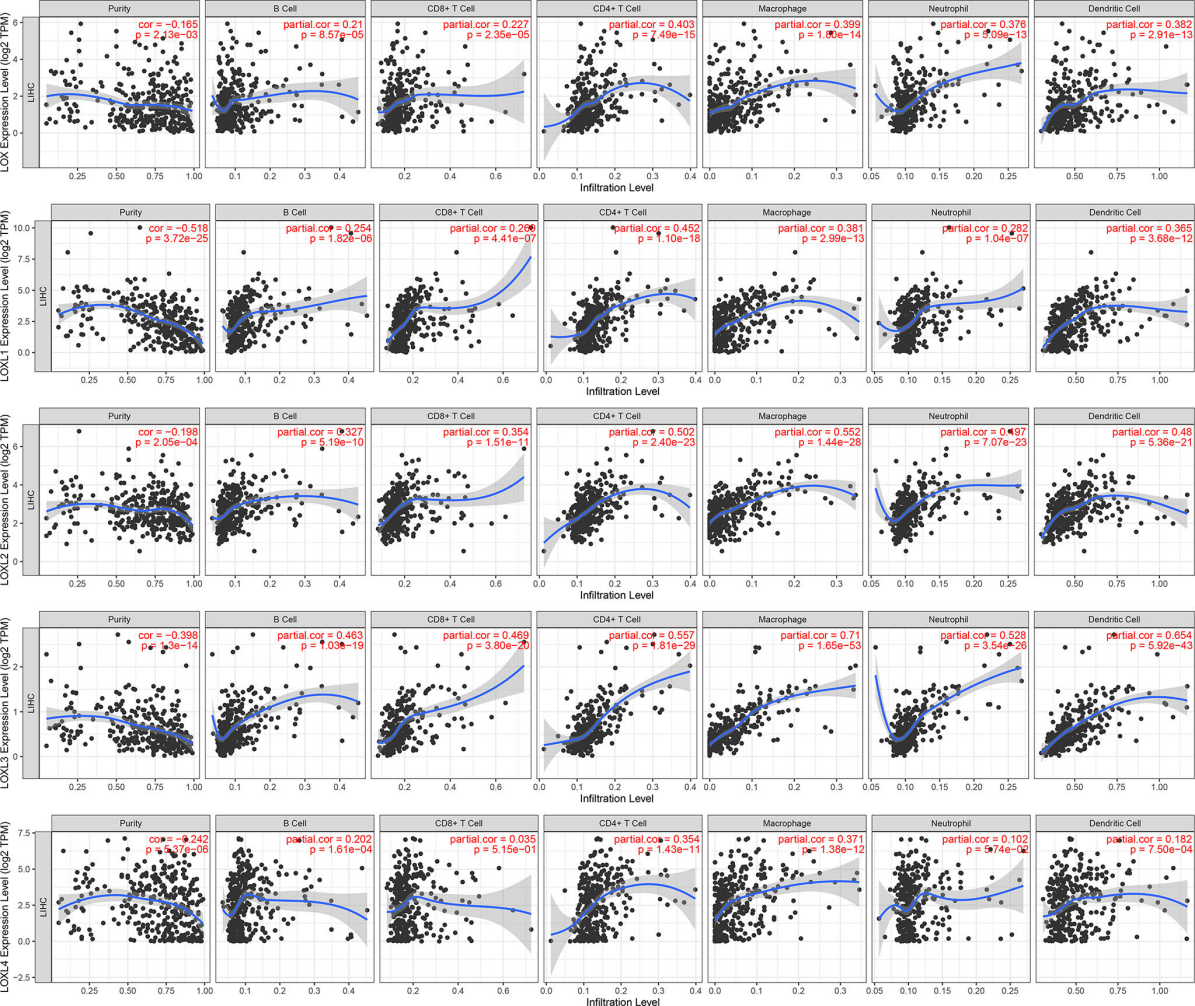
Correlations between differentially expressed *LOX* family members and immune cell infiltration in liver cancer (TIMER).

**Figure 10:**
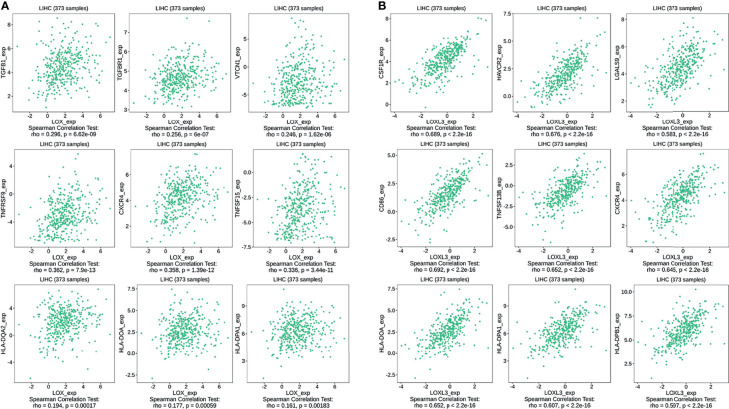
Associations of the *LOX* and *LOXL3* expression level with immunomodulators in LC from TISIDB database. **(A)** Immunomodulators that are highly correlated with *LOX;* **(B)** Immunomodulators that are highly correlated with *LOXL3*.

The TIMER database was utilized to investigate the association between *LOX* family members and immune cell infiltration, as immune cell level correlates with the proliferation and progression of cancer cells (**Figure 9**). The expression of each *LOX* family member was positively correlated with the infiltration of B cell, CD8+ T cells, CD4+ T cells, macrophages, neutrophils, and dendritic cells (DCs). Among them, Macrophage and CD4+ T Cells demonstrated the strongest positive correlation. In addition, the Cox proportional hazard model showed that B cells (*p*=0.031), CD8+ T cells (*p*=0.036), macrophages (*p*=0.027), and DCs (*p*=0.004) were significantly associated with adverse clinical outcomes in LC patients (**Table 2**). The association between immunomodulators and *LOX* family members with poor prognosis, namely, *LOX* and *LOXL3*, were then further explored. The top three immunoinhibitors associated with *LOXL3* were CSF1R, HAVCR2, and LGALS9, whilst the top three immunostimulators correlated with *LOXL3* were CD86, TNSF13B, and CXCR4. MHCs associated with *LOXL3* were HLA-DOA, HLA-DPA1, and HLA-DPB1. As for *LOX*, TGFB1, TGFBR1, and VTCN1 were the most positively correlated immunoinhibitors. TNFRSF9, CXCR4, and TNFSF15 were the three most positively associated immunostimulators. However, for MHC molecules, their associations were relatively low, with HLA-DQA2, HLA-DOA, and HLA-DPA1 as the top three molecules (**Figure 10**).

**Table 2:** The Cox proportional hazard model of *LOX* family members and six tumor-infiltrating immune cells in liver cancer (TIMER), and Number of genes that are positively and negative correlated with *LOX* family members.

	coef	HR	95%CI_l	95%CI_u	p.value	sig	Number of positively correlated genes	Number of positively correlated genes
B_cell	-7.875	0	0	0.484	0.031	*		
CD8_Tcell	-5.339	0.005	0	0.714	0.036	*		
CD4_Tcell	-4.127	0.016	0	11.792	0.220			
Macrophage	5.800	330.240	1.925	56641.590	0.027	*		
Neutrophil	-0.054	0.947	0	54322.082	0.992			
Dendritic	5.245	189.571	5.129	7006.334	0.004	**		
*LOX*	0.087	1.091	0.919	1.296	0.321		13085	6837
*LOXL1*	-0.159	0.853	0.716	1.015	0.073		12853	7069
*LOXL2*	0.019	1.020	0.742	1.401	0.905		13393	6529
*LOXL3*	-0.022	0.979	0.446	2.148	0.957		13866	6056
*LOXL4*	0.073	1.076	0.959	1.207	0.213		11983	7939

^*^p < 0.05, **p < 0.01.

95%CI_l: Lower limit of 95% Confidential Interval; 95%CI_u: Upper limit of 95% Confidential Interval.

### Co-Expression Network and GSEA Analysis of Each Member of *LOX* Family in LC

**Figure 11:**
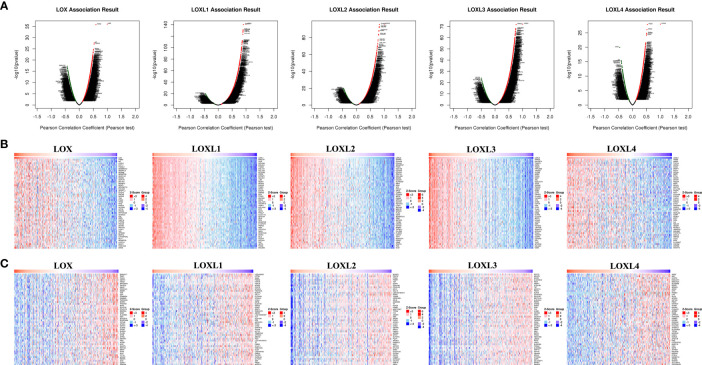
The co‐expression genes with *LOX* family members in LC from the LinkedOmics database. **(A)** The whole significantly associated genes with *LOX* family member distinguished by Pearson test in LC cohort. **(B)** Top 50 genes positively related to *LOX* family member in LC showed by heat maps. **(C)** Top 50 genes negatively related to *LOX* family member in LC showed by heat maps. Red represents positively linked genes and blue represents negatively linked genes.

**Figure 12:**
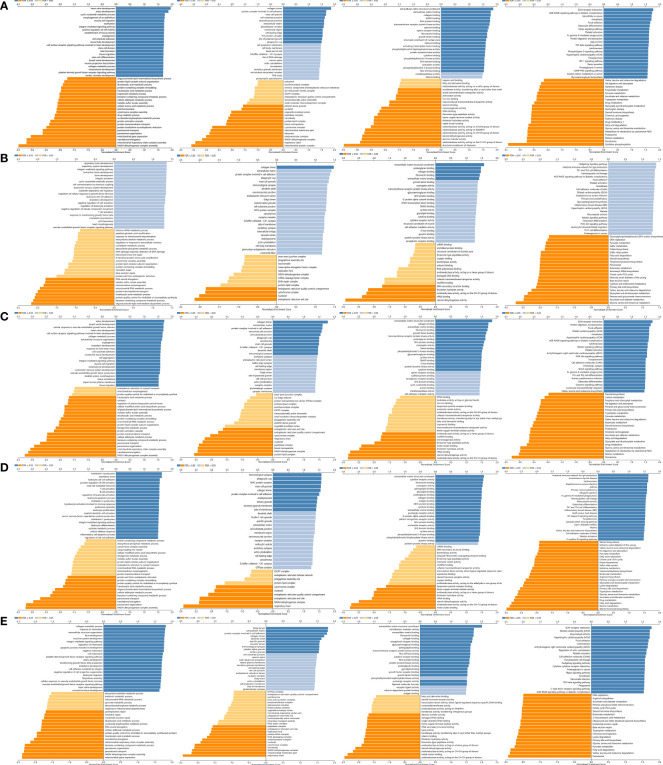
GO annotations and KEGG pathways of LOX family and their associated genes in LC cohort: **(A)** results of *LOX*; **(B)** results of *LOXL1*; **(C)** results of *LOXL2*; **(D)** results of *LOXL3*; **(E)** results of *LOXL4*.

For each member of *LOX* family, more genes (dark red dots) are positively correlated than negatively correlated (dark green dots) (**Figure 11** and **Table 2**). GO term annotation of co-expressed genes of each member of *LOX* family as well as KEGG pathway analysis were shown in **Figure 12**. These results showed a wide range of influence of *LOX* family expression network in LC.

### Meta-Analysis of the Prognosis of *LOX*, *LOXL2* and *LOXL4* in LC

Based on the search strategy, four studies (28–31) investigating *LOX*, *LOXL2* and *LOXL4* were included for the meta-analysis, while no potential literature on other *LOX* family members were found. We combined the results of our bioinformatics analysis from the TCGA with those retrieved in the database and obtained the HR values. One study (29) provided results regarding lower expressed *LOXL2* compared with higher expressed *LOXL2*, therefore, the HR was transformed using the formula new HR=e^(-ln HR) to convert the result to the OS of higher expressed *LOXL2* compared to lower expressed *LOXL2*. This resulted in the new HR of 1.761 (95% CI: 1.215-2.551).

**Figure 13:**
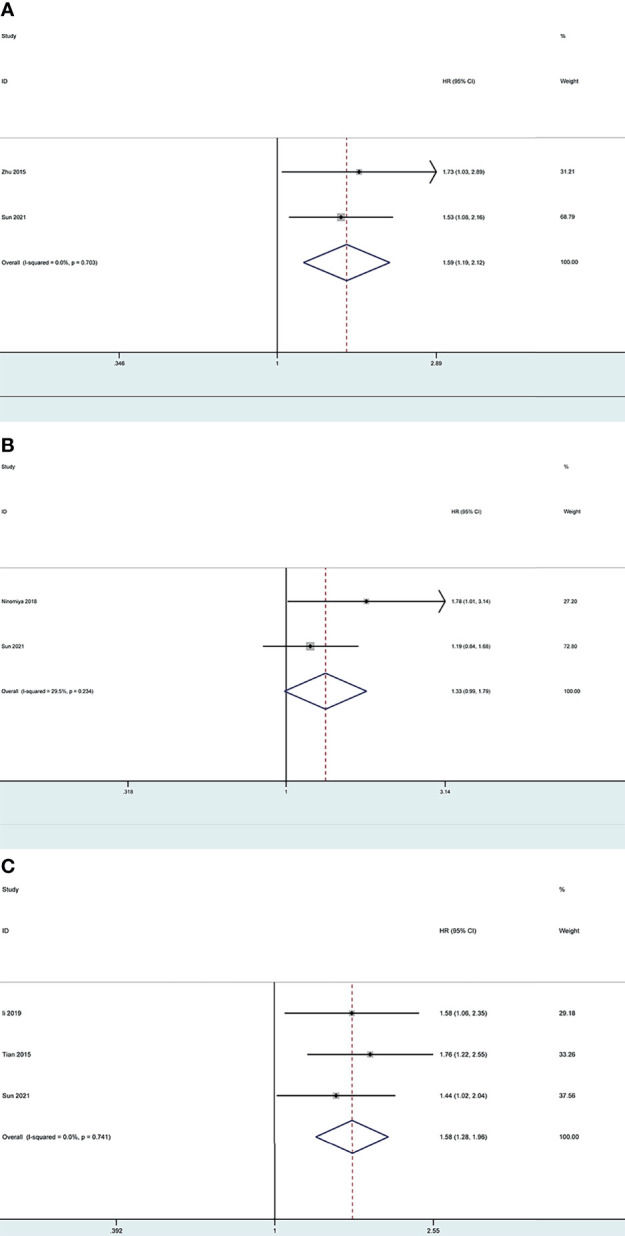
Forest plot of the prognosis of *LOX, LOXL2* and *LOXL4* for LC patients: **(A)** Forest plot of overexpressed *LOX*; **(B)** Forest plot of overexpressed *LOXL2*; **(C)** Forest plot of overexpressed *LOXL4*.

The pooled results revealed that overexpression of *LOX* and *LOXL4* were associated with worse OS of LC patients (HR: 1.59, 95% CI: 1.19-2.12, *I^2 =^* 0%; HR: 1.58, 95% CI: 1.28-1.96, *I^2 =^* 0%), while the association between overexpression of *LOXL2* and OS of LC patients showed no statistical significance (HR: 1.33, 95% CI: 0.99-1.79, *I^2 =^* 29.5%) (**Figure 13**). Sensitivity analysis indicated stable results of this meta-analysis.

## Discussion

As a common malignancy with the third leading cause of cancer-related mortality (1), LC risk is influenced by various environmental and genetic factors (4–6). Previous studies have demonstrated that *LOX* is highly expressed in the fibrous lamina propria in the small intestine, stomach, and liver, as well as other tissues that contain elastic fibers and fibrillar collagen (16). *LOX*, *LOXL, LOXL2, LOXL3 and LOXL4* were to be in intracellular locations, perinuclear regions and intranuclear locations, and are secreted to exert their functions, such as extracellular enzyme for initiating covalent cross-link formation in collagen fibrils (15, 19, 32–35). After secretion, *LOX* family members oxidase crosslink collagen and elastin (19, 36). *LOXs* were found to be involved in various physiological or pathological pathways, both in extracellular modulation and intracellular signaling (32). Studies have found *LOX* family members to be involved in carcinogenesis and tumor metastasis by formation of mature extracellular matrix at the secondary site, FAK activation, and promotion of angiogenesis (19–22). Overexpressed *LOX* was found to promote angiogenesis (23, 25), and expression level of *LOXL2* was higher in HCC than in non-tumor tissue (26). *LOXL4* was found to increase the risk of invasion and metastasis of LC *via* various mechanisms such as angiogenesis and through its involvement in creating an immunosuppressive microenvironment (25, 27, 28). However, studies on the roles of *LOLX1* and *LOXL3* in liver cancer are limited (25). As the role of *LOX* family members in LC remains inconclusive, this bioinformatic study was conducted to analyze the expression, mutation, prognostic value, and functional enrichment of *LOX* family in LC.

We found that all five members of *LOX* family are higher expressed in LC tissues than in the normal tissues and their overexpression are positively correlated with each other, which is consistent with previous findings that the expression of *LOX*, *LOXL2*, and *LOXL4* are upregulated in HCC (25). A previous study found a 30-fold increase of *LOXL1* level in a liver fibrosis model (24). However, the role of *LOXL3* in LC was not yet clear (25), and our results provided evidence that not only *LOX1* but also *LOXL3* is highly expressed in LC. As shown by the cBioPortal analysis, 5.16% of LC patients were found to have genetic mutation of *LOX* family members, and the mutation rate of *LOXL2* was the highest. Further analysis of ROC curve showed that the AUC of *LOXL2* was above 0.9 with a PPV of 0.994, indicating its potential role in diagnosis. These findings are consistent with those reported by Wong et al, in which the AUC of *LOXL2* to distinguish non-HCC and HCC patients was 0.896 (26). Therefore, *LOXL2* is a good candidate for a diagnostic marker in LC, especially HCC.

Based on the analyses through various tools, high expression of *LOX* and *LOXL3* was found to predict worse prognosis. This proves the previous hypothesis of upregulation of the *LOX* level as a predictive sign for HCC, proposed by Lin et al. (25). *LOX* gene, located at chromosome 5q23.1, is consist of a variable N-terminal domain and a highly conserved C-terminal domain (25). *LOX* itself is an extracellular, matrix-embedded protein that plays an essential role in the cross-linking of the collagen fibrils and the deposition of insoluble collagen fibers (37, 38). Previous studies indicated that *LOX* overexpression induced the Epithelial-Mesenchymal Transition (EMT) (39). In addition, Yang et al. proved that the overexpression of *LOX* activated the angiogenesis partially through increasing the VEGF and enhancing the tube formation ability of endothelial cells in tumor initiating cells (TICs)-enriched HCC, and *LOX* inhibitor β-aminopropionitrile (BAPN) reverses the angiogenesis (40). Zhu et al. also found that the proliferative, migratory. and invasive abilities of HCC cells were attenuated, and the expression of vascular endothelial growth factor (VEGF) was decreased by the silencing of *LOX*, through the p38 mitogen-activated protein kinase (MAPK) signaling pathway (30). *LOX3*, located at chromosome 2p13.1, plays an important role in remodeling the cross-linking of the structural extracellular matrix (ECM) of fibrotic organs such as the liver (25, 41). It was also shown that higher expression of LOXL3 was regulated by TGF-β in gastric cancer (42). However, studies on the biological function of *LOXL3* in HCC are still limited (25, 39, 43). Previous literature on the prognostic role of *LOXL3* in LC patients was also minimal. Therefore, meta-analysis on *LOXL3* was not conducted. Nevertheless, the result of this analysis added new evidence that *LOXL3* could be potentially used a prognostic biomarker in addition to *LOX*. A nomogram based on *LOX*, *LOXL3*, and other predictors were developed which can help predict the mortality risk for an individual LC patient. Moreover, given their negative impacts on the survival in LC patients, *LOX* and *LOXL3* may also serve as potential therapeutic targets. Although the ULCAN and TIMER did not verify the worse prognosis associated with *LOXL4*, our result based on TCGA data indicated the potential clinical significance of *LOXL4* for worse outcome. As part of the *LOX* family, *LOXL4* gene is located at chromosome 10q24.2 (25). The *in vitro* study suggested that TGF-β might induce *LOXL4* upregulation in several different HCC cell lines, and *LOXL4* mediated cell-matrix adhesion and cell migration in HCC *via* upregulation of Src and FAK phosphorylation (43). Although *LOXL4* is an important extracellular protein, the HCC cell migration was promoted more by the intracellular *LOXL4* (43). In contrast, other study revealed that 5-azacytidine (5-aza-CR)-mediated overexpression of *LOXL4* reactivated wild-type p53 and promoted cancer cell death, thus suppressing the development of HCC cancers, which might indicate an improved clinical outcomes of HCC patients (28, 44). These complicated and even contradicting mechanism of *LOXL4* in HCC might partially explained the inconsistent findings of its role in HCC prognosis from TCGA, TIMER and ULCAN database.

Further exploration of potential drugs associated with *LOX* and *LOXL3* in LC by using Coremine Medical found 30 drugs. The drug that demonstrated the strongest interaction was aminopropionitrile. β-aminopropionitrile (BAPN), obtained from a natural source, was the first compound found to have inhibitory effect on *LOX* (45). A previous study suggested the potential therapeutic value of BAPN for liver metastasis in gastric cancer (46). Another animal study demonstrated antifibrotic effect of BPAN through reducing collagen fiber bundles and *LOX* level, which indicates its potential role in attenuating the development of liver fibrosis (47). It was also found that that BAPN acts by reversing the angiogenesis that was activated by the overexpression of *LOX* (40). Although quinone and copper were found to be potential interacting drugs, they are more likely to be identified because their own function and roles in the *LOX* proteins. Quinone is part of the redox cofactor of *LOX*s, which is a functional group in the catalytic domain of *LOX* proteins (48). *LOX* family members also contain a conserved copper-binding site in the C-terminal half of the protein (49). Copper binding to key histidine residues facilitates the formation of quinone-contained redox factor which in turn leads to the oxidase activity (48, 49). Therefore, quinone and copper can be potential research targets in the future to explore any potential practical use or potential use as therapeutic target. In addition, other drugs found through the exploration, such as cetuximab, bleomycin, cisplatin, paclitaxel, were known to have anti-cancer effects in various types of cancer, including LC, and anti-fibrosis effects in other diseases such as pulmonary fibrosis (50–59). Their exact roles and effects in LC may need to be clarified in future studies.

Exploration of the PPI network of *LOX* family and their top interactors found that these genes are primarily related to extracellular structures and functions. GO enrichment and KEGG pathway analysis of these genes also found they are mostly involved extracellular functions and structures. This is not surprising as it is well known that members of *LOX* family contribute to structural integrity and increased tensile strength by their catalytic activity, and exert roles in remodeling the cross-linking of the structural extracellular matrix (ECM) of fibrotic organs such as the liver (25). In addition, LOX family members are involved in scavenger receptor activity, oxidoreductase activity, and copper ion binding. Multiple scavenger receptor cysteine-rich (SRCR) domains exist n *LOXL2* and *LOXL3* (60, 61). As *LOX* family are copper-dependent amine oxidases (25), it is not unexpected that oxidoreductase activity and copper ion binding are involved. Further analysis *via* LinkedOmics database also identified a significant amount of co-expressed genes associated with each *LOXL* family members, and found that these co-expressed genes are also largely involved in extracellular and functions and structures, or participate in human tissues that contain elastic fibers, fibrillar collagen, and organs with a great amount of fibrous lamina propria.

The growth and metastasis of tumor cells depend on a complex tumor microenvironment (TME) (62). TME comprises of cells of hematopoietic origin, such as lymphocytes and myeloid cells, cells of mesenchymal origin, including mesenchymal stem cells, endothelial cells, adipocytes, fibroblasts, and myofibroblasts, and the ECM (63). ECM is a complex network providing structural support, biochemical reagents and biomechanical signals for the growth of cancer cells, and it consists of multiple components, including collagen, integrin, laminin, fibronectin, glycosaminoglycans, matrix metalloproteinases (MMP) and secreted cysteine-rich acidic proteins (64). Further analysis on the relationship of *LOX* family members and tumor-infiltrating immune cells in LC found positive correlations between the infiltration of B cell, CD8+ T cells, CD4+ T cells, macrophages, neutrophils, and DCs and all *LOX* family members. Moreover, the infiltration of B cells, CD8+ T cells, macrophages, and DCs were associated with worse outcomes. Immune cell infiltration in HCC under different conditions, such as bile acid-mediated immune cell infiltration (65) and TP53 mutations (66), have been investigated in the past. However, evidence on the associations between *LOX* family members and tumor-infiltrating immune cells in LC is limited. In addition, immunomodulatory drugs are under development for various conditions and have been approved in recent years for certain tumors such as multiple myeloma (67, 68). Therefore, we explored and identified a list of immunoihibitors, immunostimulators, and MHC molecules that are positively correlated with *LOX* and *LOXL3*, the two *LOX* family members with poor prognosis. These immunomodulators could be potential immunotherapeutic targets. The immune environment is thought to be critical in tumor progression and may even play a crucial role in different treatments for cancers, including chemotherapy, radiotherapy, and especially immunotherapy (69–71). Our findings suggest that there is a significant role of the *LOX* family in the tumor microenvironment. Therefore, comprehensive studies on the association of tumor-infiltrating immune cells, as well as immunomodulators, and *LOX* family in LC are needed.

It is well known that tumor heterogeneity relies on the TME, including both the cancer cells themselves and different types of immune cells and the surrounding stroma. TME closely correlates with the response to immunotherapy and the prognosis in multiple cancers (72). TME tends to be involved in the immunosuppression and drug resistance, resulting in less satisfactory responses to immunotherapy. In addition, immune checkpoint blockade (ICB) relies on restoring the function of T cell to eliminate tumors (70). Moreover, as part of the adaptive immune resistance, tumor cells could upregulate the immune checkpoint gene expression to suppress T cell activity that eventually leads to immune escape (73). Thus, our findings of positive association between CD8+ T cells and CD4+ T cells, as well as other immune cells, and *LOX* family members suggest that ICB and other immunotherapy could have a promising potential in LC treatment as high expression of *LOX* family members in tumor tissues facilitates immune cells infiltration, which could induce the immune response exerting the antitumor efficiency. It would particularly helpful to investigate compounds target on immunoinhibitors and immunostimulaters identified in our study. As demonstrated in our study immunoinhibitors, CSF1R, HAVCR2, and LGALS9, were found to be associated with *LOXL3*, while TGFB1, TGFBR1, and VTCN1 correlated with *LOX*. CSF-1R plays critical roles in regulating tumor-associated macrophages in TME, and targeted inhibition of the CSF-1/CSF-1R signal axis has broad application prospects in immunotherapy of malignant tumors (74). Pexidartinib is an orally administered small-molecule tyrosine kinase inhibitor that selectively inhibits CSF1R, and is currently being assessed for other types of cancer (75). Another kinase inhibitor, Derazantinib, also found to have activity against CSF1R and is under investigation for cholangiocarcinoma (76). Their potential use in LC also deserves further exploration. HAVCR2, also known as TIM-3 and CD366, enhances T cell inhibition and apoptosis and immune-suppressive activity of Tregs (77). Antibodies against HAVCR2 disrupt the binding of the ligands to HAVCR2 are under investigation as a potential combination partner of anti-PD-1/L1 therapy (78). Previous study also found that HAVCR2 receptor limits T-cell responses by interacting with LGALS9 (79). A recent study also demonstrated that chemoradiation could induce increased expression of PD-L1 and LGALS9 in gastric cancer (80), however, whether similar result can be found in LC needs further study. TGF-B1 is a potent inhibitor of T cell growth, partly by inhibiting IL-2 expression and secretion by T cells themselves (81), and interestingly, it can also affect anti-tumor T cell responses by downregulating MHC molecules on the surface of tumor cells. Despite its critical roles, the development of TGFB1-targeting therapies has not been progressed well, probably due to concern of severe toxicities that could arise from blocking tumor suppression exerted by TGF-β1 at early stages of tumorigenesis as TGFB1 exerts potent cytostatic and pro-apoptotic activities in pre-malignant cells (82, 83). In addition, blocking TGFB1 activities on normal cells outside of the TME may also lead to toxicities (82). Nevertheless, certain antibodies are still under investigation. For example, studies on Fresolizumab, a fully human monoclonal IgG4 antibody that neutralizes mature TGFB1, were conducted for malignant pleural mesothelioma, melanoma and renal cell carcinoma (82). Galunisertib, another TGFBR1inhibitor, was found to have 16% of objective responses in glioblastoma patients with no serious treatment-related toxicities (82, 84). Another clinical trial for pancreatic cancer patients showed that combination of chemotherapy (gemcitabine) and galunisertib was associated with increased survival compared to chemotherapy alone (85), and it is now also tested for combination with anti-PD-1 antibodies (82). In addition, a new TGFBR1 kinase inhibitor called vactosertib, currently tested in early-stage clinical trial for several cancer types (83). VTCN1, also known as B7S1, is also a negative regulator of tumor immunity by various mechanisms such as dampening the anti-tumor Th1 responses (86). Recently, an early-stage clinical study of FPA150, an antibody targeted on B7S1 and other the anti-B7x family members, was started for patients with advanced solid tumors to assess preliminary efficacy of FPA150 alone or in combination anti-PD, as well its safety, tolerability, pharmacokinetics, and pharmacodynamics (87). In addition to immunoinhibitors, the immunostimulators CD86, TNSF13B, and CXCR4, were found to be associated with *LOXL3*, while TNFRSF9, CXCR4, and TNFSF15 correlated with *LOX*. CD86, also known as B7-2, is an immune checkpoint molecule of B7 family and binds to CD28 and Cytotoxic T-Lymphocyte Antigen 4 (CTLA-4). Interaction of CD86 with CTLA-4 inactivates T lymphocytes, causing the escape of tumor cells from the immune system. Therefore, immunotherapy using CTLA-4 antibodies might promote T cell activation to help eliminating tumor cells (88). Ipilimumab, an CTLA-4 antibody, is currently approved by Food and Drug Administration (FDA) for HCC treatment in combination with nivolumab. In addition, Tremelimumab, fully human immunoglobulin G2 monoclonal antibody directed against CTLA-4, is also under investigation for HCC treatment (89). TNSF14B, also known as B cell-activating factor of the TNF family (BAFF), together with its receptor, BAFFR, are important in early B-cell homeostasis and regulatory T-cell function (90). BAFF inhibitors have been tested for certain diseases. For instance, belimumab, a fully human monoclonal antibody against BAFF, has been shown to have a modest effect for active systemic lupus erythematosus (SLE), and another BAFF inhibitor Blisibimod is also under investigation for SLE (90, 91). Tabalumab is another BAFF inhibitor, and has been evaluated as a combined therapy with bortezomib for multiple myeloma (90). However, the roles of BAFF inhibitors in HCC still yet to be explored. TNFRSF9, also known as CD137, a surface glycoprotein belonging to a member of the tumor necrosis factor receptor superfamily (TNFRSF). It is expressed on activated T cells that have encountered cognate antigen, activated NK cells, and mature DCs (92). Two clinical trials have been being initiated for two anti- TNFRSF9 monoclonal antibodies urelumab (BMS-663513) and utomilumab (PF-05082566) (93, 94). TNFSF15, also called TNF-like molecule 1A (TL1A), is expressed on multiple immune cells such as DCs and B cells. It binds to DR3 receptor, leading to cell apoptosis by activating the caspase cascade through interaction with TRADD and FADD, and the activation of multiple cell survival signaling pathways including NF-kB, STAT3, JNK, p38 MAPK and ERK (95, 96). TNFSF15 can also suppress endothelial cell proliferation and angiogenesis through the binding of DR3, and this was verified in a mouse xenograft tumor model (97, 98). Moreover, TNFSF15 also can be induced in T cells, macrophages, monocytes, and DCs in response to stimulation with immune complexes, Toll-like receptor ligands, inflammatory cytokines, and T-cell receptor activator (99). Current studies mostly focus on the role of TNFSF15 in inflammatory diseases such as SLE and psoriasis (100), its potential roles in HCC yet to be further investigated. Apart from the above immunostimulators, the CXCR4 was associated with both LOX and LOXL3. It is expressed on various pro- and anti-inflammatory immune cells, especially in macrophages and T cells (101). Multiple drugs targeted on CXCR4 have been under investigation (102). AMD3100, also known as plerixafor (Mozobil), was the FDA-approved CXCR4 antagonist used for peripheral blood stem cell transplantation, but its clinical use in LC and other solid tumors is limited due to its poor pharmacokinetics and toxic adverse effects (102, 103). Recently, other CXCR4 antagonists have been developed. For example, BPRCX807 has been experimentally validated in different HCC models (103), and it deserves further investigation. The MHCs, HLA-DOA, HLA-DPA1, and HLA-DPB1, positively associated with LOXL3 all belongs to MHC Class II molecules (104). MHC class II molecules were found to be expressed by antigen-presenting cells, including antigen-presenting cancer-associated fibroblasts (apCAFs) (105, 106). Therefore, these positive association of the MHC Class II molecules might be indirect evidence of apCAFs in HCC, and drugs targeted on these apCAFs might have potential therapeutic values and future studies are needed for further clarification.

This bioinformatic study also acknowledges some limitations: First, as all data were retrieved from online databases, the results still need to be validated with other experiments and cohorts. Second, as this study was mainly aimed at exploring the potential diagnostic, prognostic, and therapeutic values of the *LOX* family members in LC patients, the details of their mechanisms were not comprehensively explored. Third, most of the samples on the online databases were HCC, therefore their values on other types of LC still need further investigation. Fourth, meta-analysis found that *LOXL4* was associated with poor OS, while results from TIMER and UALCAN did not yield the same conclusion. However, only two studies on the survival effect of overexpressed *LOXL4* were found, therefore, more studies are urgently needed to validate its effect of the prognosis for LC patients.

## Conclusions

This bioinformatics analysis investigated the expression levels, diagnostic and prognostic values, genetic alterations, PPI network, functional enrichment, tumor microenvironment factors, and potential mechanisms of *LOX* family members in LC. Our results found that all *LOX* family members are overexpressed in LC tumors, and *LOXL2* is good candidate as a diagnostic marker. *LOX* and *LOXL3* are associated with poor prognosis and carry potential as therapeutic targets. The effect of *LOXL4* on survival remains equivocal and prompts more studies. The infiltration of a variety of immune cells and a list of immunomodulators were positive correlated with *LOX* family members. These results highlight the need to explore the roles of *LOX* family in the tumor microenvironment and their potential as immunotherapeutic targets.

## Methods

### Analysis of *LOX* Family Expression Levels

The expression levels of *LOX* family members between LC and normal tissue were first compared by using the Wilcoxon rank sum test, and visualized by ‘ggplot2’ package of R software version v3.6.3 (The R Foundation for Statistical Computing, 2020). p < 0.05 was considered statistically significant. Data extracted from The Cancer Genome Atlas Liver Hepatocellular Carcinoma (TCGA-LIHC) database (https://portal.gdc.cancer.gov/), and Log2 transformed FPKM (fragments per kilobase exon-model per million reads mapped) were used.

To further verify the expression levels of the 5 members of *LOX* family between LC tissues and adjoining normal tissues, the difference in transcriptional levels were assessed using students’ t-test through the UALCAN online tool (http://ualcan.path.uab.edu/analysis.html), in which a statistically significant value was defined as *p*-value < 0.05 (107). These findings were then verified through Tumor Immune Estimation Resource (TIMER) (https://cistrome.shinyapps.io/timer/), an online tool based on data of more than ten-thousand tumors from 32 types of cancer (108, 109).

The optimal discriminate cut-off point between the high and low expression groups was evaluated by the receiver operating characteristic (ROC) curve and area under the curve (AUC) values for overexpressed *LOX* family members, with data obtained from the TCGA-LIHC database. Log2 transformed FPKM were used for downstream analyses. ROC curve was created by using pROC and ggplot2 packages of R software.

### Analysis of Prognostic Value of *LOX* Family Expression in LC

The prognostic value of *LOX family* expression was first explored based on the TCGA-LIHC data with Log2 transformed FPKM. We applied the Kaplan-Meier (KM) survival analysis with log-rank test to compare the survival difference between high expression group and low expression group. The KM curves, with p-values and hazard ratio (HR) with 95% confidence interval (CI), were generated by log-rank tests and univariate Cox proportional hazards regression. These calculations were performed using R software with ‘survminer’, and ‘survival’ packages. The results were verified by through the UALCAN online tool (107) and TIMER (108, 109).

A predictive model based on TCGA-LIHC data was also established to predict the mortality risk based on overexpressed members of *LOX* family and all other potential predictors (110–112). A nomogram using ‘rms’ and ‘survival’ R packages was developed, based on multivariate Cox proportional hazards analysis for predicting the 1,3,5-year overall survival. A graphical representation of potential predicting factors was provided by the nomogram to calculate the risk of mortality for an individual patient. In order to assess the discriminatory performance of the model, C-index was also calculated (112–114).

### Analysis of Genetic Mutations of *LOX* Family in LC

Five datasets, including “TCGA, Firehose Legacy”, “RIKEN, Nat Genet 2012”, “AMC, Hepatology 2014”, “INSERM, Nat Genet 2015”, and “MSK, Clin Cancer Res 2018” were applied to analyze gene mutations of *LOX* family members *via* cBioPortal (http://www.cbioportal.org/). cBioPortal is a comprehensive web resource providing visualization, analysis, and download of large-scale cancer genomics data sets (115). The correlation of *LOX* family members with each other was calculated by analyzing mRNA expressions (RNA sequencing [RNA-seq] version (v.)2 RSEM) in the cBioPortal online tool for Liver Hepatocellular Carcinoma (TCGA, Firehose Legacy). Pearson’s correction was included. TIMER was also used to verify the correlation of *LOX* family members using the Correlation module (108, 109).

### Exploration of Potential Drugs That Are Interacted With *LOX* Family in LC

Potential drugs that interact with members of the *LOX* family and demonstrated significant difference in expression and survival between LC and normal tissues were investigated through text mining. Coremine Medical (http://www.coremine.com/medical/) was used to visualize the connections among genes and pathways (116, 117).

### Analysis of Interaction of *LOX* Family Members in LC

Protein-protein interaction (PPI) network analysis was performed on differentially expressed *LOX* family members and their most significantly interacted proteins *via* STRING online database (https://string-db.org/) (118) and GeneMANIA (http://www.genemania.org) (119).

### GO Enrichment and KEGG Pathway Analysis of *LOX* Family Members

Functions of *LOX* family members and their top 20 most associated genes identified from GeneMANIA (119) were analyzed by Gene Ontology (GO) and Kyoto Encyclopedia of Genes and Genomes (KEGG) in the DAVID database (https://david.ncifcrf.gov/summary.jsp) (120, 121). GO enrichment analysis predicted the function based on biological processes (BP), cellular components (CC), and molecular functions (MF), while KEGG analysis determined the related pathways of *LOX* family members and their associated interactors. The results of GO and KEGG analyses were visualized by the Bioinformatics online tool (http://www.bioinformatics.com.cn) (122, 123). KEGG online web tool (http://www.genome.jp/kegg/), an integrated database for biological interpretation of genome sequences and other large-scale molecular datasets, was also used to verify crucial pathways (123–126).

### Immune Cell Infiltration of *LOX* Family Members in LC

The infiltration of different immune cells and their clinical impact were assessed through TIMER, an online tool for comprehensive molecular characterization of tumor-immune interactions (108, 109). Plots were generated using the Gene module in TIMER, through which we analyzed the correlation between the expression of *LOX* family members and immune infiltration level in LC. Cutoff value of Cor >0.2 and p<0.05 were used to determine a significant correlation (127, 128). To further explore the interactions between immune system and *LOX* family members that are associated with poor prognosis, the TISIDB database (http://cis.hku.hk/TISIDB) was used. TISIDB is a web portal for analyzing immune system and tumor interaction, including nearly one thousand reported immune-related anti-tumor genes, etc., and immunological data gathered from seven public databases (129–131). Here, TISIDB was used for exploring the immunomodulators associated with *LOX* family members in LC.

### Association Analysis of Each Member of *LOX* Family and GSEA Analysis

The LinkedOmics (http://www.linkedomics.org/login.php) is an online tool with multi-omics data from 32 types of cancer based on TCGA (132). *LOX* family members were screened from the TCGA-LIHC cohort by choosing HiSeq RNA as platform and RNAseq as data type in both search dataset and targe dataset. The genes associated with each member of *LOX* family member were explored through the LinkFinder module, and the correlation of results was tested by the Pearson correlation coefficient and presented respectively in volcano plot and heat maps. Function module analysis of GO and KEGG pathways were explored by the gene set enrichment analysis (GSEA) in the LinkInterpreter module.

### Meta-Analysis of the Prognosis of Overexpressed *LOX* Family Members in LC

A meta-analysis was performed to verify the results of OS of overexpressed *LOX* family members in LC. Two authors (S. Mao and Y. Chen) independently searched the potential articles related to *LOX* family members and LC published until May 2021 *via* the Cochrane Library, PubMed, Web of Science and CNKI (Chinese National Knowledge Infrastructure). To find all eligible literature, the following search strategy was used: *(LOXL1* OR *LOXL2* OR *LOXL3* OR *LOXL4* OR *LOX* OR lysyl oxidase like 1 OR lysyl oxidase like 2 OR lysyl oxidase like 3 OR lysyl oxidase like 4 OR lysyl oxidase) AND (liver cancer OR hepatocellular carcinoma OR LC OR HCC). Chinese phrases replaced the English terms in the CNKI database. Before conducting this study, we consulted the Preferred Reporting Items declared by the Systematic Review and Meta-Analysis (PRISMA) (133). Then, the strength of associations between *LOX* family members and OS in LC was evaluated by calculating the combined HRs with the corresponding 95% confidence interval (CI). *I^2^* statistics were used to assess the degree of heterogeneity across the incorporated original studies (134). If *I^2^* > 50%, the random-effects model was used to estimate the HR to account for heterogeneity; otherwise, the fixed-effects model was applied (135). In addition, we performed sensitivity analysis by switching between the random-effects model and fixed-effects model and observing for significant differences in the results (136, 137). The above statistical analysis was performed using STATA 15.1. statistical software (Stata Corp., College Station, TX).

## Data Availability Statement

The datasets presented in this study can be found in online repositories. The names of the repository/repositories and accession number(s) can be found in the article/supplementary material.

## Author Contributions

YZ and CS designed the research study. CS, YC, and SM selected and collected the data. CS, YC, SM, and WG analyzed the data. CS, YC, SM, and NK wrote the manuscript. ZZ participated in the statical analysis. SK, YW, WG, YC, JT, CB, NM, YH, CC, and QZ provided critical opinions and revised the manuscript. All authors read and approved the final manuscript.

## Funding

The Training Program Foundation of Youth Scholars by the First Affiliated Hospital of Anhui Medical University (2020kj24).

## Conflict of Interest

The authors declare that the research was conducted in the absence of any commercial or financial relationships that could be construed as a potential conflict of interest.

## Publisher’s Note

All claims expressed in this article are solely those of the authors and do not necessarily represent those of their affiliated organizations, or those of the publisher, the editors and the reviewers. Any product that may be evaluated in this article, or claim that may be made by its manufacturer, is not guaranteed or endorsed by the publisher.

## Abbreviations

LC, Liver cancer; HCC, Hepatocellular carcinoma; LOX, lysyl oxidase; LOXL, lysyl oxidase-like; FAK, focal adhesion kinase; BAPN, β-aminopropionitrile; ECM, extracellular matrix; SRCR, scavenger receptor cysteine-rich; TME, tumor microenvironment; ECM, extracellular matrix; MMP, matrix metalloproteinase.
